# History of a Case of Lymphadenosis

**Published:** 1885-12

**Authors:** John Meredith

**Affiliations:** Wellington, Somerset


### Clinical Records.

### HISTORY OF A CASE OF LYMPHADENOSIS.

By JOHN MEREDITH, M.D., Wellington, Somerset.

IN the beginning of the year 1877, W. H. consulted me about a swelling on his neck, size of a hen's egg. He was 21 years of age, and was engaged as an assistant in one of the large warehouses of the metropolis. He was frequently employed in the underground rooms of the establishment, in which the atmosphere was stuffy and close. He had been in the same employ during the previous year. He led, as was stated, "a rather fast life" during his stay in London; but he considered himself healthy and strong, until towards the end of 1876.

When he visited his home in the country, that Christmas, he was noticed to be a little anæmic; tongue, pale, furred, and slightly tremulous; quick, irritable pulse. He, however, returned to his duties after his few days' holiday; but he said that he was "not up to the mark." On the 9th of February, 1877, while having a bit of play with one of his fellow-assistants, he felt a sharp pain on the right side of his neck, owing to a sudden turn of his head during the tussle. Next morning a painful swelling was noticed behind the angle of the jaw. It remained for a few days, then seemed gradually to disappear, but did not really do so completely.

After this the right ear became painful, an abscess formed and burst, seemed to recover for a while, then became painful, formed and burst again; and this happened several times during the spring of 1877. The swelling behind the angle of the jaw began to increase in size. The patient had to give up business in town and return to his home. This occurred about the end of April. He had been taking various forms of iron tonics before he came home, and continued doing so afterwards. The tumour was movable, and felt hard; no fluctuation; it was evidently glandular. From this time on, I had the care of the case. I took the temperature occasionally, and found it one to three degrees above normal; pulse rapid, from 100 to 120. Dr. Andrew Clark examined the case early in June, and said he feared the presence of a tubercular element, but that the lungs at that time were all right.

On the 12th of June it was noted that he was getting thinner, and had occasional nocturnal sweatings. The lungs seemed healthy. There was no perceptible indication of tubercle. The condition of the heart was normal. The patient had, except at times, a fair appetite, and his bowels acted regularly. There was no sign of disease about either the head or spine, nor in connection with the generative organs.

The family history was considered good; no history of scrofula or phthisis in it; and the young man had been well cared for and lived well.

The chief thing now was the tumour in the neck. It had been increasing in size among the deeper tissues, as well as outwardly. It was seen bulging in the pharynx, and compressing the nasal orifice. He became subject to fits of nausea and vomiting at times, and felt sick at once when the lower edge of the tumour was pressed.

A glandular swelling formed on the left side after a while. The date upon which this was noticed first is not mentioned. On the 25th of June the following note was made: Patient lying in bed; pulse, 100; temperature, normal. Lungs and heart seem healthy. The tumour on the right side remains about the same in regard to size. It appeared to press less on the pharynx than it did. There is a great deal of muco-purulent discharge, and at times nausea and vomiting. The tumour on the left side, behind the ear, is increasing in size.

July 18th. The patient was by the seaside at Teignmouth, for a change. I saw him there on this date, before he left his bed. He complained of feeling very weak. He had been taking chloral for the relief of pain, and it had disagreed with him. He was often taking some anodyne or another. His pulse this day was 120; temperature, 99.5°; respiration, normal; tongue, moist. Complained much of a "thickening in the throat," with muco-purulent discharge adhering to the fauces; but he had not expectorated much of it lately. Suffered from paroxysms of pain in the neck and head: the pain seemed to spread from the glandular tumour in the neck, sometimes very severe at night.

The state of the chest was normal, save that respiration murmur appeared feeble on the right side, and the cardiac sounds louder; percussion sound normal in both lungs. There was no sinking in of the chest-walls.

The tumours on each side of the neck further increased in size, and were tender to the touch. I could detect no fluctuation at the time. He was able to take a fair quantity of food, notwithstanding the suffering.

Bromides with hyoscyamus were given, with the view of affording relief from this. The patient's strength was day by day decreasing.

July 22nd he returned home. On examining him that evening I found decided dulness on percussion over the left side of the chest, with decreased respiratory sound everywhere.

July 28th. The dulness had disappeared; respiratory murmur, feeble; pulse, 112 to 120; temperature, 99.5°. Four days later his condition seemed better. He had rallied, said he felt better, able to take a good amount of food, and did not require chlorodyne or anything of the kind to procure rest and sleep. Suffered from fits of gloomy despondency. The urine was examined this day, and found of normal character.

On the 10th of August, Mr. John Gay, of Finsbury Place, London, saw the patient in consultation (Mr. W. Edwards acting on my behalf, owing to my absence), and it was decided to incise both tumours, as each had now a fluctuating area.

The incisions gave vent to a quantity of serous fluid. It was expected it would have proved to be a sort of thin, curdy substance. The growth on the right side had an opening into the pharynx, which was distinctly felt behind the right tonsil. Through this orifice the glandular tumour had discharged portions of its contents.

After the incisions had emptied all that could escape, a considerable quantity of glandular growth remained, which had not melted down.

Note made on the 14th of the month is to the following effect: "There has been a great amount of discharge from the tumour on the right side, and there is a slough to come away; the discharge is mainly serous. The pulse last evening was 140; temperature, 103°. Still, the breathing seemed normal; in no way very hurried. There was nothing particular to observe in regard to the condition of the lungs, except that vocal resonance appeared somewhat increased. This morning the pulse was 136, and temperature 100.3°. The patient faints upon the slightest exertion; says that he does not feel the pain in the throat and the swellings as much as he did before the latter were incised."

At this time he was labouring under the combined exhaustion of rapid pulse, abnormal temperature, and great discharge from the glandular enlargement of the neck. Against this had to be placed the fact that he was able to take a good amount of nourishment, including beef-tea and other articles. I scarcely expected he would be able to live many days, judging from his then condition.

On the 17th of August he seemed better; been improving for three days. Pulse, 120; temperature of the body, normal. Discharge from the right tumour more purulent in character than heretofore; sleeps better, and said he was able to swallow his food with comparative ease. Very little discharge from the tumour on the left side.

August 24th. A severe rigor set in on the afternoon of that day. Temperature rose to 104°, and the pulse to 160 or thereabouts; I was not able to count it correctly. The gland on the right side of the neck had a bag-like patch of fluctuation: the contents burst during the night, and discharged through the opening previously made. The edges of the sore through which the discharge escaped were red and prominent, and a piece was clipped off and microscopically examined, with the result of showing that it was only "proud flesh," with no sign of malignancy.

On the 27th of August he rallied, and appeared to have got over the exacerbation which set in three days before. On the evening of the 24th there was a good deal of bilious vomiting, and bilious matter was passed in the stools. The two succeeding nights were restless, chiefly from rheumatic pains in the right shoulder; but, as stated, the patient seemed better on the 27th. He insisted upon those about him giving him alcohol in some form or another, and appeared to be taking more than was good for him.

The tumour on the right side of the neck seemed as if it was breaking down by suppuration; but there was little change in the character of the swelling on the left side. There was nothing to describe in the way of change until the middle of September, when the suppurative discharge from the right tumour gave place to a discharge of a serous character; and on the 23rd this stopped, and the orifice closed; but it was soon found out that it imprisoned some fluid, which readily fluctuated.

After all the discharge, there was no perceptible decrease of the size of the glandular swelling, beyond that now it did not cause much inconvenience through pressing on the pharynx. The state of the left tumour remained much the same, discharging some semi-serous fluid every day.

On the 25th of September the pulse was 130 and upwards; temperature, 99°. There was a cheerful brightness about the patient's face and the tone of his voice that day. The condition of the lungs remained about the same; vesicular breathing, feeble; appetite good; bowels and kidneys acting regularly.

His blood, when examined under the microscope, showed a great deficiency in the number of the red corpuscles. The patient was weak and thin. At this time he was taken to stay at a house on the Blackdown

Hills; but no particular improvement resulted from the move. At the end of October his condition was as follows: Pulse, 120 and sometimes higher; temperature, 99.5°; breathing, normal. There was a peculiar condition of the chest: no complaint of pain: woodeny sound on percussion: tubular respiration: hardly any vesicular breathing anywhere.

Under Mr. Gay's instructions, the glandular swellings were injected with a dilute solution of hyd. pernit., and ten-minim doses of tinct. mur. fer. given internally thrice daily; but this was followed by an attack of headache every time it was tried. The injection of the solution induced some suppuration in the left tumour; but none would take place in the one on the right, even when tried more than once. No marked change otherwise.

On the 29th of October the percussion-sound seemed clearer over the chest: there was a slight cough and some expectoration; but no râles could be detected, and there were none at the date of the next entry, one week later. The patient was then able to take food fairly well, for him; was always sleepy in the daytime, occasionally wakeful in the evenings; getting thinner and thinner; clubbing of the finger-nails very clear. The discharge from the tumours thin and serous again. Occasionally he experienced twinges of pain spreading from the right tumour down the arm and side.

On the 22nd of November the pulse was 130 and over; temperature, 100°. For a few days before this the pulse was as low as 112, with normal temperature, but it was only for a short time. The tumour appeared to soften. Had a cold, with some cough and expectoration; still, I was not able to detect râles in the chest. When asleep, he had an irregular stridulous breathing; was weak, and slept much. I painted the tumours with tincture of iodine twice a week, but with no advantage to the patient. He took a fair quantity of cod-liver oil for a time; then it was exchanged for koumiss, which in three days had to be discontinued, as it caused biliousness. All medicines were stopped for a time; then resumed in the form of cod oil and fer. redact. The patient, however, often rebelled against anything of the kind, and he was not pressed to take any when not disposed to do so. The "spasms," as he described the attacks of pain, extending from the left cervical tumour down the shoulder and arm, harassed him frequently: sometimes they lasted an hour or more; at other times, but a few minutes.

There was little to notice from day to day. The glandular swellings on each side of the neck bulged out to the level of the shoulders.

On the 24th of December it was noted that the general emaciation of the body was most marked. Appetite capricious: some days very little food was taken, and the patient was gloomy and despondent; at others, tolerably bright and hopeful. The neuralgic spasms connected with the left side became more frequent, and lasted for hours. The swelling had an angry appearance. The "spasms" recurred if food was not supplied punctually. At times the patient swooned away in those attacks of pain, and remained in a semi-conscious state for a long time; and was occasionally carried from his chair upstairs to his bed in that condition. His pulse at this period ranged from 116 to 140; the temperature almost always a little above normal.

An abscess formed over the left clavicle. The pressure of the left tumour on the pharynx increased, and gave rise to hawking expectoration.

The tumour on the right side seemed quiet. By the 11th of January, 1878, the gland above the left clavicle was much enlarged—formed a glandular cyst—skin over it red and thin. As it caused discomfort, I tapped it by trocar and canula, and drew off five fluid ounces of clear straw-coloured fluid; no pus or blood. The fluid was of neutral reaction, highly albuminous, and contained a great deal of chlorides.

Other glands of the neck had swollen before this one, and their contents had been withdrawn by incisions, as was done in the first instance by Mr. Gay, and in every case the character of the fluid was similar to the above. In regard to one sample, Mr. Gay wrote that "the fluid had an alkaline reaction, and contained a large number of blood globules, some of which were exceedingly pale, or rather white, and others moderately red. There was an intermixture of others of every intervening shade of colour. There were few lymph corpuscles, and a considerable quantity of albumen. A blood-clot separated from the fluid, which was very tough, and consisted of white and red blood globules and some few lymph cells. It was toughened by the albumen."

At this time there was very little discharge from the glandular enlargement on the right side; what was, seemed to have been clear glandular secretions, similar to what was poured out by the one on the left.

Lymph-secreting, apparently, was the work of the system, which, owing to the waste of it, was producing rapid emaciation of the body. The blood of the young man was being diverted from its flesh-forming function by the inordinate activity of the hypertrophied cervical glands, and thown away in the form of serum.

What could have been the exciting and sustaining cause of this?

January 17th. The urine had been examined at intervals since the commencement of the illness, but it was always found in normal condition. On this date its sp. gr. was 1015, with some earthy phosphates; but there was nothing special to note in this respect.

The patient was not entirely confined to bed until a couple of days ago. He slept a great deal, in short snatches; the expectoration from the throat was excessive, and prevented long periods of rest. The spleen and liver seemed of ordinary size, no pain or tenderness about them. There was no enlargement of the glands anywhere, except in the cervical regions.

February 10th. The patient remarked that morning that it was just that day twelve months he had that "crick" in the neck, which began all his troubles. As to progress, there was little to note. The discharge from the right side stopped some time previous to the above date, and had not returned. The glandular enlargement on this side spread forward under the chin: it was not painful, except when handled.

There were two glandular openings on the left side, discharging serous fluid. Glands in the axillæ were noticed to be enlarged. On the 6th, pain was complained of at the base of the left lung, acute when coughing; and there was a space indicating marked dulness on percussion, and moist râles when examined by stethoscope, with marked difficulty of breathing. The patient's body was extremely emaciated, and complete faintness followed upon any exertion, even trying to change position in bed. He became rapidly worse; the pneumonic area of the left lung increased, until by the 12th there was only a small margin in front which gave anything like normal resonance. No complaint of pain; complained much of the pressure of the tumours on the gullet preventing deglutition; hardly a drop of fluid could pass. Death resulted on the evening of the 13th. The intelligence continued unaltered, to all appearance, until within a couple of hours of the heart's action stopping.

Two or three days before death, I noticed that the margin of the glandular sores in the neck were no longer red: they had become pale; the red blood, what there was of it left, being, no doubt, diverted into the left pulmonary space, blocking it.

Examining the body after death, I found no appreciable enlargement of the spleen or liver; no glandular enlargement anywhere, except in the axillary and cervical regions: these last were hard, and dipping graspingly into the deeper parts of the throat. No further examination was permitted. Had there been such an opportunity, I have little doubt I should have found that the cervical tumours compressed and interfered with the course of the pneumogastric nerve, accounting for the pains complained of; and discovered growths disseminated in the substance of the lungs, giving rise, probably, to the peculiar resonance and respiratory murmur mentioned, and inducing in the left lung the pneumonic condition which ended fatally.

As for treatment, everything that could fairly be suggested was tried, but nothing proved of any particular use. One distinguished medical practitioner to whom I spoke about the case, at the Manchester Meeting of the British Medical Association, said: "No matter what is done in these cases, they always end fatally; but try calcüchlor." I did so, but it did no good.

I have not had so marked a case of Lymphadenosis as this to attend to either before or since; and the literature of the disorder, although considerable, helps very little towards elucidating its natural history. Looking at the case with the assistance of information which has come to light since, I am disposed to class Lymphadenosis among the filth diseases, and consequently preventible.

In Dr. Gower's article on this disease (Hodgkins), in *Reynolds's System of Medicine*, the enumeration of the "earliest symptoms" points irresistibly, according to my thinking, to the chief cause being persistent exposure to some marked insanitary condition or other. That organs take on excessive action and increase in size, to compensate for shortcomings on the part of other organs, is a recognised fact in medicine; but that they take on excess of action and persist in it, not because of other organs' defect, but owing to some vitiating, noxious influences, often not comprehended, is, perhaps, not equally well recognised. This form of action seems like a desperate strain to overcome a difficulty, so as to secure again the equable level.

Many of us, when exposed to unpleasant odours, especially such things as sewer gas, experience excited action of the salivary glands, and saliva flows freely. The sore throats frequently resulting from such exposure are attended by thickening of the cervical glands; and enlargement of the tonsils is common in such cases, likewise of the salivary glands.

The rush of saliva which sanitary inspectors experience in visiting ill-smelling localities is but the desperate strain of excretory organs to repel an uncongenial intruder; and he whose sense of taste and smell is not quick on such an emergency is to be pitied, and there are many such. The intrusion may result in possession being taken in some form or another of blood poisoning; a condition being then established when "a crick," or some apparently minor action, is sufficient to determine the form of a fatal disorganising force.

Trousseau's view is that the disease at first is local, started by local irritation; and Dr. Gower's, that it may be local and constitutional in origin; and the particulars they adduce amount to nothing decidedly clear and specific.

Although I am not able to give an account of the condition of the various organs of my patient's body by a regular *post-mortem* examination, still, I can describe the hygienic conditions under which he lived.

At the commencement of the paper I mentioned that W. H. was said to have led a rather "fast life" when in London; but there was no history of gonorrhœa, chancre, or syphilis, and there was no reason to suspect his ever having had anything of the kind. The "fast life," whatever it was, could have been nothing vitally injurious. Still, opinions may differ on this. But, like others, he had to live for hours each day in ill-ventilated places when in the discharge of his work.

Next to this must be considered the sanitary influences under which he lived at his father's house in the country. This house, although to all outward appearance it seemed everything that could be desired as a family dwelling—roomy, well-furnished, and cleanly,—yet it possessed serious sanitary defects: 1. The water used was bad, not fit for use, yet was used. It was derived from a well, around which was a network of brick drains, in constant use. 2. Sewer air entered the house through the overflow of the rain-water cisterns under the kitchen floor. 3. Through the lead pipe of an upstairs watercloset, of the old D-trap and iron-container sort: the pipe was eaten through, and had many small holes. 4. The pantry sink-pipe smelt badly when the plug was out: it passed, like the closet one, into the main drain, without any syphon or other form of check. 5. A small wash-hand place in the hall was connected with the drains in the same way, and smelt badly at times unless the plug was fixed in. 6. Under the back stairs of the house, and close to a couple of rooms in which the family lived most of their time, a bad smell was often noticed, and no one was able to say where it came from. However, after some years the house changed owners; and in September, 1884, assisted by the present possessor, I was able to make a thorough examination of the house, when the state of things just enumerated was brought to light; and we further found that the smell under the stairs was caused by sewer gas passing along a rat-hole from a drain, through the wall near the foundation, up under the floor into the place, taking its share with the rest in poisoning the air of the house.

The defects, I have no doubt, existed in 1877 8, and had done so for years before, although their deleterious influence was not suspected.

Cases of illness were always slow in recovering in the house, and its occupants frequently appeared languid, and often ailing. Thus, poor W. H. had the odds against him in every way. It might be asked why improvement did not appear when the patient was away on the Black-down Hills or at the seaside. To this I would reply that both the glands and the system generally had been constrained into a bad form and habit—through long immersion in an impure atmosphere—from which they could not break off.

